# An event-oriented database of meteorological droughts in Europe based on spatio-temporal clustering

**DOI:** 10.1038/s41598-023-30153-6

**Published:** 2023-02-23

**Authors:** Carmelo Cammalleri, Juan Camilo Acosta Navarro, Davide Bavera, Vitali Diaz, Chiara Di Ciollo, Willem Maetens, Diego Magni, Dario Masante, Jonathan Spinoni, Andrea Toreti

**Affiliations:** 1 European Commission, Joint Research Centre (JRC), Ispra, Italy; 2 ARCADIA SIT, Vigevano, Italy; 3 Technische Universiteit Delft, Delft, Netherlands; 4 ARHS Developments, Milan, Italy; 5 Dipartimento di Ingegneria Civile e Ambientale, Politecnico di Milano, Milan, Italy

**Keywords:** Hydrology, Natural hazards

## Abstract

Droughts evolve in space and time without following borders or pre-determined temporal constraints. Here, we present a new database of drought events built with a three-dimensional density-based clustering algorithm. The chosen approach is able to identify and characterize the spatio-temporal evolution of drought events, and it was tuned with a supervised approach against a set of past global droughts characterized independently by multiple drought experts. About 200 events were detected over Europein the period 1981-2020 using SPI-3 (3-month cumulated Standardized Precipitation Index) maps derived from the ECMWF (European Centre for Medium-range Weather Forecasts) 5th generation reanalysis (ERA5) precipitation. The largest European meteorological droughts during this period occurred in 1996, 2003, 2002 and 2018. A general agreement between the major events identified by the algorithm and drought impact records was found, as well as with previous datasets based on pre-defined regions.

## Introduction

Drought is a systemic natural disaster, with important repercussions in almost all sectors of societies, ecosystems, and economies^1^. Recent estimates of drought impacts over Europe are in the order of €9 billion per year, with climate change projections indicating an increasing threat^2^. Our lack of knowledge on the mechanisms that control droughts’ onset, evolution, and recovering, hampers our capability to make predictions^3,4^, thus limiting our ability to understand how major droughts will evolve in space and in time.

While drought is widely recognized as an event-oriented phenomenon, it is rarely treated as such^5^. The joint spatial and temporal dynamics of drought have been investigated only in a few research studies^6,7^, and in most cases not integrated into operational monitoring systems. However, the need to base drought policies on the best possible description of these events suggests that incorporating such aspects into early warning systems^8^ is of primary importance. The World Meteorological Organization (WMO) Resolution 9 (CG-17)^9^, constitutes a further push towards an event-based approach in cataloguing natural disasters, with the introduction of the concept of a universal unique identifier (UUID) for high impact extreme weather, water and climate events.

The key role of a transboundary database of drought events is also emphasized by the need to unambiguously attribute sparsely-collected reported losses to a specific drought event^10^, an inherently challenging task in areas such as Europe where drought information is often available only in regional and/or national reports^11^.

Previous studies generally focused on building drought event databases at regional^12^ and national^13^ scale, and even continental^14^ and global^15^ scale studies mostly relied on aggregated data over pre-determined areas (i.e. water basins, countries or regions). While this approach is valuable in allowing the comparison of past events occurring over the same area, it obviously misses the large spatial connectivity that characterise major events and that distinguishes drought from other natural hazards.

A few attempts at boundless analyses of droughts are available in the literature^6,16,17^, but these studies mostly focused on major droughts, and their outcomes depend on a pre-determined cluster model parameterization often derived from past experience. Three common traits of these studies are the lack of an explicit contextualization of the adopted parameterisation in the framework of the common classification of drought (i.e. meteorological, agricultural, hydrological, etc.), any form of tuning against an independent reference dataset, and the use of a simple Boolean drought classification to discriminate areas under drought.

In this study, we aim at developing an event-oriented database of meteorological droughts that overcomes the above-mentioned limitations by: (i) adopting a flexible clustering algorithm that can adjust to different drought definitions, and (ii) tuning the clustering algorithm on a reference dataset specifically built for short- and medium-term meteorological droughts. The implemented approach is a generalized three-dimensional clustering algorithm^18^, which introduces a set of parameters that allow to adapt the degree of spatio-temporal aggregation of drought cells. To overcome the lack of a widely available reference dataset (for the tuning of the algorithm), a expert-based assessment of reference events was chosen and a supervised tuning approach was performed. Global scale estimates of drought events are obtained, but a special focus is given to droughts over Europe, where the outcomes are analyzed against data on impacts as well as previous studies using pre-defied regions.

## Results

### Tuning of the clustering method

Starting from the expert-based characterization of the 38 global past events, a set of meteorological drought events between 1981 and 2020 are here identified from a time series of SPI-3 maps according to the generalized three-dimensional clustering algorithm. This approach searches for areas with a high-density concentration of cells under drought without any a-priori definition of the spatial or temporal domains. The size, number and shape of the clusters depend on a set of six parameters (*L*, *p*, *e*, *k*, *A*, *R*, see the “Methods” section) to be tuned following a trial and error supervised approach, starting from the values commonly adopted in the relevant scientific literature^6,16,17^, and moving throughout a pre-determined set of parameterisations defined according to the model sensitivity analysis^18^. In particular, according to a previously performed sensitivity analysis^18^, the commonly used two-dimensional application of the contiguous drought area (CDA) represents a lower boundaries for most parameters (i.e. largest clusters). Thus, the starting set of parameters (set = 0) is based on the CDA, where all the contiguous drought cells (using a 8-neighborhood rule) are considered part of the same cluster (within the searching window of 3 × 3 cells), and drought conditions are defined by a simple threshold value (SPI-3 = − 1.5). Each set of tuning parameters was evaluated according to: an overestimation factor *f*_over_ (ratio between the cells of the 3D cluster that falls inside the reference and the total size of the cluster); and an underestimation factor *f*_under_ (fraction of cells under drought inside the reference that are not included in the given 3D cluster).

According to the results summarized in Table 1, the CDA (*L* = 3 and *p* = 0) with Boolean threshold (*k* = 1.5 and *e* = 1000, i.e. all cells with SPI-3 under − 1.5 are classified as drought) approach (set = 0) performs the best in minimizing the underestimation of the expert-based events (*f*_under_ = 90.1%). However, this parameterization also has a clear tendency to overestimate the size of the expert-based event (*f*_over_ = 80.0%, much worse than any other sets of parameters). This is likely due to the fact that CDA considers a cluster even if just one additional cell in the surrounding is considered under drought, as well as due to the absence of distinction between cells with different drought severity (Boolean threshold).

**Table 1:** Results of the tuning procedure for different sets of parameters of the clustering algorithm.

Set	*L*	*p*	*k*	*e*	*A*	*R*	*f*_over_ (%)	*f*_under_ (%)	*f*_avg_ (%)
0	3	0	1.5	1000	100	3	80.0 ± 29.5	**90.1 ± 19.1**	85.1 ± 25.2
1	3	0.25	1.7	8	100	3	93.3 ± 16.1	85.9 ± 20.7	89.6 ± 18.8
2	3	0.25	1.7	8	200	3	96.9 ± 6.2	87.0 ± 18.4	**91.9 ± 14.5**
3	3	0.25	1.7	8	200	5	94.0 ± 18.3	82.6 ± 24.4	88.3 ± 22.1
4	5	0.30	1.7	8	200	3	90.3 ± 18.2	88.2 ± 20.5	89.2 ± 19.3
5	3	0.25	1.7	8	250	3	**97.3 ± 6.0**	83.6 ± 22.7	90.5 ± 17.9

Values in bold highlight the maximum for each metric. See “Methods” for a detailed description of each parameter and metric.

*L* size of the searching window in space, *p* fraction of cells in the surrounding to detect a core cell, *k* mid-point of the logistic curve, *e* steepness of the logistic curve, *A* minimum size of the 2D clusters, *R* size of the searching window in time.

Following the sensitivity analysis, we searched for an improvement in *f*_over_ (and as a consequence a maximization of the average of *f*_over_ and *f*_under_, *f*_avg_) by introducing two modifications to set = 0: (i) the use of a logistic weighting function in lieu of the Boolean threshold (*k* = 1.7 and *e* = 8), (ii) by strengthening the constraint on the number of minimum neighbouring cells (*p* = 0.25, a minimum of 4 cells in the surrounding need to be under drought to define a core point) (set = 1). Other small tuning of either the *p* parameters or the weighting function did not return any meaningful changes in neither *f*_under_ nor in *f*_over_ (not reported). A further increase in both *f*_under_ and *f*_over_ is obtained by increasing the minimum cluster size (*A*) from 100 to 200 cells (set = 2), but a further increase in *A* (i.e. to 250 cells) caused an improvement in *f*_over_ but at the expense of *f*_under_ (set = 5). The introduction of an asymmetric search window – larger in either space (*L* = 5, set = 4) or time (*R* = 5, set = 3) – does not seem to introduce any clear improvement in the performances. While other small modifications of the parameters were tested, no significant changes from the sets reported in Table 1 were obtained.

We assumed as optimal parameterization set the one that find the best compromise between minimizing the underestimation (splitting the drought events into multiple sub-events) and the overestimation (merging multiple distinct events into a single cluster) of the reference events (i.e. maximizing *f*_under_ and *f*_over_). This can be simply quantified by the average of the two metrics, *f*_avg_, which clearly shows how the optimal solution is represented by the one identified as set = 2 (maximum *f*_avg_).

**Figure 1:**
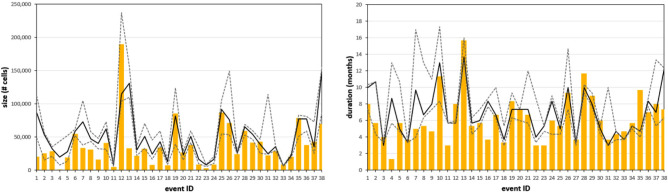
Comparison between the results of the selected parameterization (orange bars) and the reference dataset for events’ size (left panel) and duration (right panel). The black line is the reference ensemble median, while the grey dotted lines represent the 30-th and 70-th percentiles.

Data in Fig. 1 compare the outcomes of the optimal parameterization against the reference expert-based dataset, both in terms of total size of the event (left panel) and duration (right panel). These results show the overall good agreement with the ensemble median of the reference.

The modelled events remain inside the range of dispersion of the reference in most of the cases, with a slight tendency to underestimate both size and duration in agreement with the metrics reported in Table 1 (*f*_under_ = 87.0%, *f*_over_ = 96.9%). The few instances where the model overestimates the reference seem to be mostly driven by spatial overestimates (i.e. the cluster extends outside the reference area) and they occur mostly for the most recent reference events, whereas the underestimation seems to be more marked for smaller drought events (mostly concentrated at the beginning of the reference dataset, around the 80s) such as the event in Patagonia in 1989 (ID = 4). A detailed report of the type of error for each event is available in S1.

### Database of drought events over Europe

**Figure 2:**
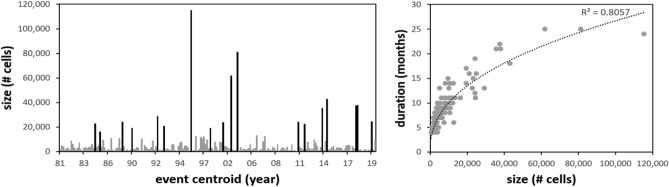
Summary of the outputs of the clustering analysis. The left panel shows the temporal distribution of the events, with the black bars highlighting the major droughts (size > 15,000 cells). The right panel shows the relationship between the size and the duration of the events, with the dotted line representing a power relationship.

Starting from the global outcomes of the optimal procedure, we focused our analysis over the pan-European domain, by extracting a subset of the identified events comprising all the clusters with a centroid lying within Europe (from upper left corner -15 E, 72 N to lower right corner 50 E, 35 N). Smaller events are removed from the successive analyses by filtering out clusters with a size lower than 500 cells and a duration of less than a month. According to these rules, a total of 198 events in the period 1981-2020 are identified (Fig. 2).

The frequency distribution of the cluster size roughly follows a power-law relationship, with 18 major events (> 15,000 cells) covering more than 50% of the sum of all drought events, and half of the events covering almost 90% of the total. The black bars in Fig. 2 (left panel) highlight these major droughts, showing a high frequency of major events in the 2010s (7 events). The strong (*R*^2^ = 0.81) power relation between size and duration of the events (Fig. 2, right panel) suggests that any consideration on the duration of the event can be also easily extrapolated from the size and vice versa; hence, the subsequent analyses will focus mostly on the latter one.

**Figure 3:**
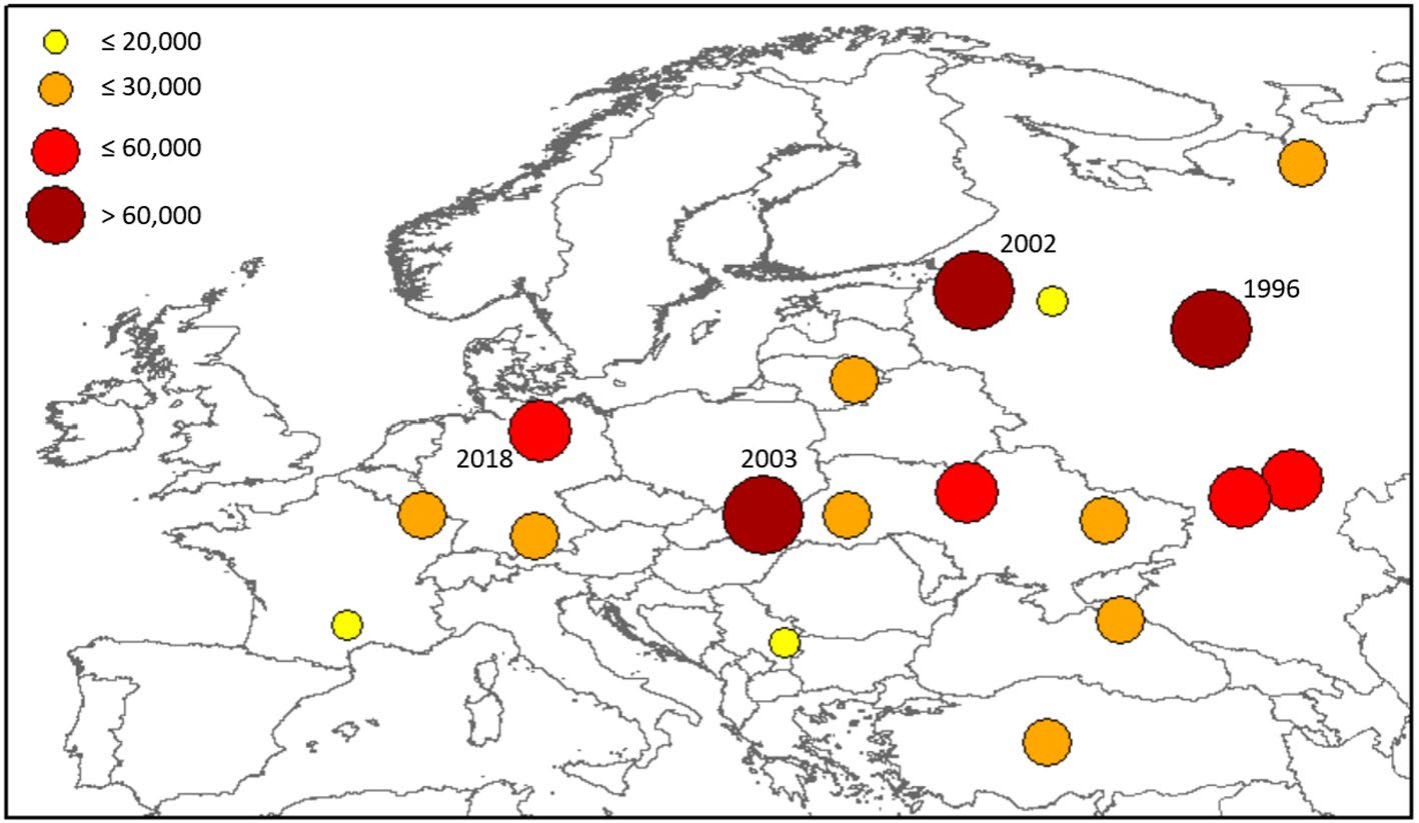
Location of the centroids of the major droughts in Europe according to the clustering algorithm. The circles are proportional to the size of the event (in number of cells).

The map in Fig. 3 shows the centroid of the major droughts, highlighting a concentration towards Central and Eastern Europe. Large size droughts in south-west Europe are limited due to the presence of natural barriers, such as the Alps, the Pyrenees and seas, which break the spatial connections (even if the largest event related to persistent atmospheric patterns induces events that spread over the barriers). This of course results in smaller but more numerous droughts over these regions. It is worth highlighting how among the largest droughts, two of them, 1996 and 2002, have the centroid located in the north-east quarter of the domain, whereas the two centred over central Europe, 2003 and 2018, are among the most referenced recent events^19–22^.

## Discussion

The interpretation of both the optimal parameterisation and the corresponding results should be carried out by keeping in mind the specific goal of building a database of short- and medium-term meteorological droughts. Additionally, to contextualize our results properly, it is relevant to stress how most of the previously published studies were often lacking any proper tuning of the parameters against an independent reference, as they mostly relied on expert knowledge or automatic procedures for their parameterization.

The obtained optimal solution is generally more conservative compared to the commonly adopted CDA approach^6,16,17,23,24^, due to the increased constraint in the searching for neighbouring cells (*p* = 0.25 corresponds to 4 neighbouring cells in contrast to the single one of CDA, *p* = 0) and the introduction of the weighting function that moderate the capability to form clusters to the less extreme negative SPIs. This result is also in line with the reference dataset adopted for the tuning, focused on short- and medium-term droughts compared to multi-year events used in other studies^17,25^.

The method also favoured a relatively large minimum threshold (*A* = 200 cells, roughly corresponding to 150,000 km^2^). Smaller values of *A* were mostly adopted in studies at continental scale^23,25^ (between 25,000 and 40,000 km^2^), and in some cases they were arbitrarily chosen based only on experience^17^. Global scale applications gravitate towards even larger values of *A*^6,16^ (from 200,000 up to 500,000 km^2^), but also had a clear objective to pick out only major droughts. In this regard, the obtained solution represents a trade-off between the desire to avoid neglecting regional-scale events and the need to minimize the effects of tenuous spatial connectivities^16^. The observed tendency to underestimate the size of the event (in both size and duration) for the smaller reference cases may be a side effect of this choice, since a smaller *A* value may be preferable to avoid the early-interruption of small droughts. In this regard, a variable *A* parameter as a function of the event size may be a potential further development of the methodology, implying the need to implement an iterative procedure to optimize this parameter. The spatial resolution of the data may also play a role in this tendency to underestimate the size of the smaller events, since coarse spatial resolutions may limit the capability of the algorithm to capture the persistence of events that are small relatively to the cells size.

The optimal parameterisation obtained in this study also depends on the initial assumptions adopted to retrieve the reference dataset, and the choice to focus on short- and medium-term meteorological droughts. As already mentioned, some of the past event-oriented studies often retrieved multi-annual droughts^17,25^, whereas our reference dataset is mostly comprised of seasonal to annual events that are commonly monitored using SPI-3 or other similar drought indicators. While the generalized cluster methodology is designed to adapt to different definitions of drought, a specific tuning would be required, based on a dedicated training dataset of events, if a different drought definition or indicator is used.

Even if the tuning performed in this study explored a reasonable range of parameterizations, following a logical sequence of trial and error tests, a more robust parameterization of the methodology may need to follow a more objective optimization strategy. In this context, the role of artificial intelligence may be explored, even if the training of such approaches usually requires an extensive reference dataset.

A major advance of this methodology is the possibility to facilitate a direct intercomparison among events recorded on different continents, since the algorithm has been tuned against a dataset of global events and the adopted parameterization is the same for all the retrieved events. This remove any subjectivity in expert-based analyses performed by different experts in different regions with various understanding of drought, as well as of a-priori constraints related to pre-determined reference regions.

To further elaborate on the outcomes of the clustering algorithm, this database of events built over Europe is compared with the data available on a European national- and regional-database of events^14^, as well as with some study cases extracted from the European Drought Impact Inventory (EDII)^11^.

Of the 12 events listed among the biggest multi-region droughts in Europe in the period 1981–2012 according to an approach based on pre-defined aggregation regions^14^, 7 are included among the 18 major droughts assessed by our clustering algorithm. These events are the southern European droughts of 1985 and 1990, the central Europe droughts of 1992, 2003 and 2011, the 1996 central and north-eastern Europe event, and the early 2001 drought in south-eastern Europe.

Two events, in 1983 and 1994, have a centroid located outside the eastern side of the subset domain, mostly comprised of a large fraction of Russia, whereas the events in the Baltic countries in 2007, the Iberian peninsula in 2005, and Aegean countries in 2007, were not included among the 18 major droughts by our algorithm, albeit they were identified by the algorithm. The latter two are also included among the cases study of EDII, hence they are discussed in more detail later on.

The 2018 event in central and northern Europe is commonly considered one of the most impactful in recent years, and it has been widely discussed in the scientific literature^21,22^. It is interesting to notice that the period 2018–2020 has been also referred to as a multi-annual drought period^17^, and that two other major droughts are indeed located within this time frame according to our algorithm. Other studies analysed the period 2014–2018 as a multi-year drought^26^, with the two major droughts in 2014 and 2015 also discussed there.

Notably, there is relatively limited literature on the two major droughts of 1996 and 2002 (see Fig. 3), even if the 1996 drought was mentioned as the largest (after the one in 1976) observed in north-western Europe^27^, and the 2002 event was reported among the major droughts of the 2000s^28^. Some entries are available in the EDII regarding these two events, mostly for central Europe in 1996 and Scandinavia in 2002, but the small number of reported impacts compared to the events in 2003 and 2018 (of similar size) may be due to the well-known limited coverage of EDII for northern and eastern countries^11^.

Direct comparisons in terms of drought extent and severity between our event-oriented database and the region-based estimates are not possible, due to the obvious differences in modelling assumptions and underlying datasets. Nevertheless, analysis of the SPI for fixed time periods (calendar years) and space (Europe) also results in the identification of the years 1996, 2018, 2003, 2002 and 2011 as the ones with the highest drought area in Europe (see S2), further validating the estimates in this study.

**Figure 4:**
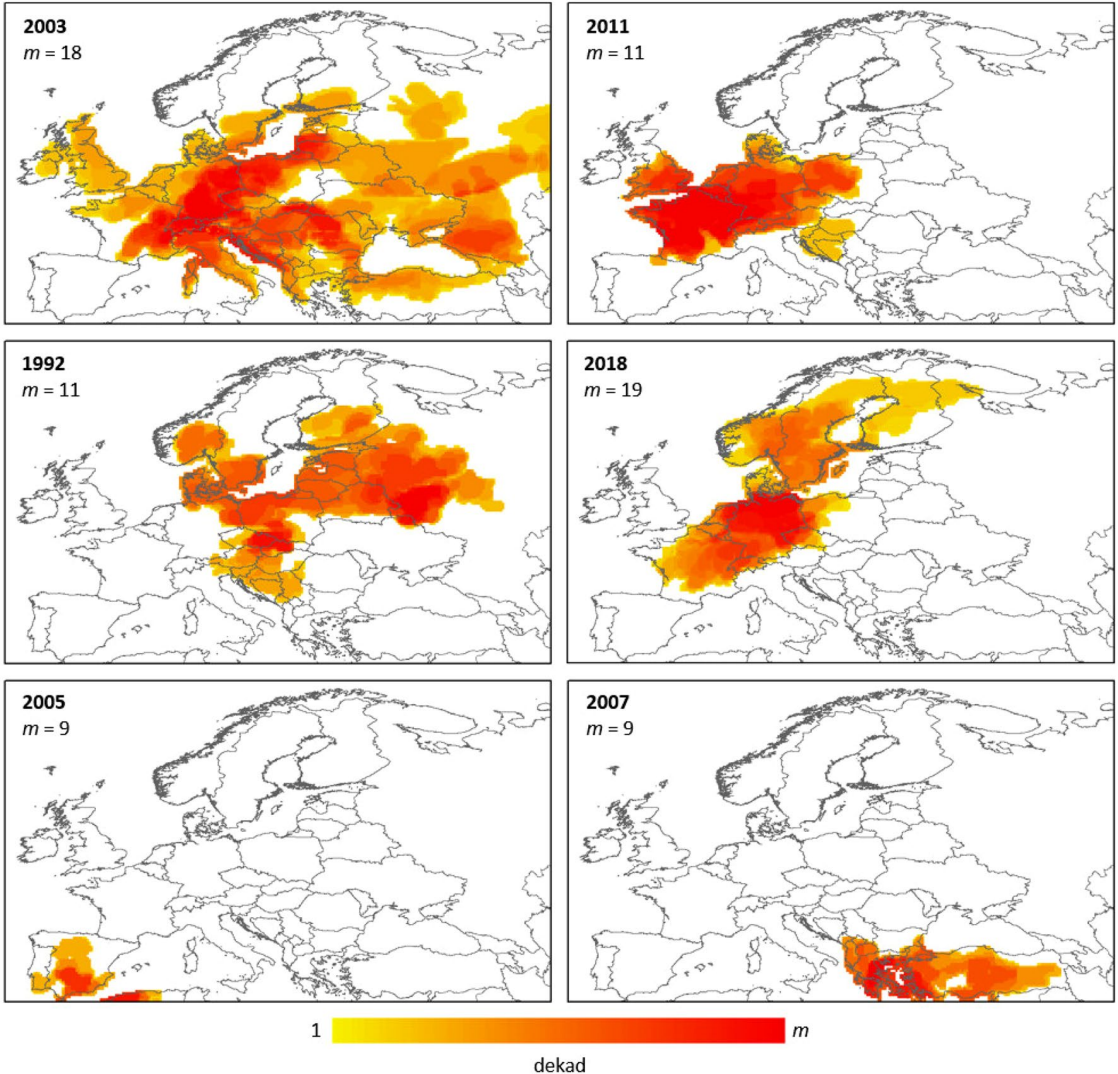
Heat-maps of some relevant droughts documented in the scientific literature and the EDII impact database. The maps represent the duration of the drought for each cell, obtained by summing along the temporal axis the clusters in all the time steps covered by the specific event. Note that even if the colour scale is the same for all the maps, the maximum value (*m*, in dekads) varies.

Many of the droughts previously discussed are also documented as part of the EDII, with some of the events discussed in terms of spatial distribution of the impacts^11^. Even though the approach of using the number of entries found in the database for each drought is not fully representative of the impacts of the event, these maps can still be used to qualitatively assess the representativeness of our outcomes. In Fig. 4 we report the heat-maps for a set of selected droughts, including both Europe-wide and regional events. Those heat-maps represent the drought duration of the event for each cell, by superimposing the cluster estimates for all the time steps covered by the event.

The drought of 2003 is correctly identified in terms of the wide spread of the event, and the location of the core of the event in Germany is also well identified. For the event in 2011, the clustering approach correctly identifies the core on France and south UK, where most of the impact reports were recorded, even if areas in Germany and eastern Europe are also included. The 1992 drought event correctly covers mostly north-eastern Europe, with numerous impacts reported in Poland and Sweden. The regional events over the Iberia Peninsula in 2005 and south-eastern Europe in 2007 are also reasonably well assessed in terms of the spatial location of the clusters. Even if not included in the EDII study, the event of 2018 is here reported as it is discussed in depth in the scientific literature. Also in this case, the extent of the event and the major focus over central and northern Europe is well identified.

While the matching between drought clusters and reported impact for these selected cases is satisfactory, it still important to stress that the largest events do not necessarily equal the most impactful, since many factors such as regional exposure and vulnerability also play a key role1. Following that, a 1:1 match between meteorological events and reported impacts is not to be expected.

Overall, the proposed approach seems to capture most of the major droughts recorded in the last forty years, removing the barriers of pre-defined aggregation regions or time windows. The structure of the clustering algorithm is well suited for a progressive application, as part of a monitoring system to track on-going events. In this regard, one major advantage of this event-oriented approach is the possibility to facilitate the use in disaster monitoring systems, such as the Global Disaster Alert and Coordination System (GDACS, www.gdacs.org), which are by nature event-oriented. In addition, the integration of a unique identification system, such as the one promoted by WMO^9^, is greatly facilitated by an event-oriented approach.

A clear outcome of the study is the dependence of the database of events from the parameterisation of the clustering approach, and how an ad-hoc tuning of the system to the given set of objectives (i.e. meteorological droughts, or multi-annual events) is crucial. In this regard, the role of artificial intelligence can be investigated to deploy objective strategies for the model tuning.

## Methods

The Standardized Precipitation Index^29^ at 3 months accumulation (SPI-3) is here adopted, since it is a common choice to characterize short- and medium-term meteorological drought conditions^30^. Precipitation data from the ECMWF (European Centre for Medium-range Weather Forecasts) 5th generation reanalysis (ERA5)^31^ were used to compute the global SPI-3 maps at 0.25 degree spatial resolution for the period 1981-2020, for roughly 10-day intervals (i.e. dekads: 3 SPI values per month)^32^.

SPI estimates require the fitting of a probability distribution on a series of multi-annual precipitation data, covering the 30-year period 1981-2010 as suggested in the WMO guidelines^33^. The gamma distribution is fitted by means of the Generalized Additive Model in the Location, Scale and Shape (GAMLSS) modelling framework^34^. Cells and periods with more than 10 precipitation values lower than 0.01 mm in the baseline are masked out, as SPI is not suitable for such dry areas (i.e. deserts).

Drought clustering is based on a generalization of the contiguous drought areas approach (CDA)^23^, following a weighted three-dimensional density-based spatial clustering of applications with noise (DBSCAN)^35^. In this approach, a three-dimensional data cube (longitude, latitude and time) is analysed by searching in a three-dimensional neighbour space a minimum data density to define a cluster. A weighing factor is associated to each data cell in order to control the capability of each cell to form a cluster, and a minimum cluster size is introduce to remove noise. This methodology is implemented through a set of six parameters, which increases the capability of the methodology to adapt to different drought definitions^18^:

- *L*: the size of the search window in space used to detect neighbouring drought cells (i.e. *L* = 3 corresponds to a 3 × 3 search window in space);
- *R*: the size of the search window in time, which may differ from the one in space to account for possible spatio-temporal asymmetry (i.e. *R* = 5 corresponds to a larger search window in time than in space if used along *L* = 3).
- *p*: the fraction of the cells within the search window that must be under drought to define a core cell (i.e. *p* = 0 corresponds to a single cell in the surrounding to define a cluster, as the minimum possible number of cells);
- *k*: centre value of the weighting logistic function (corresponding to a weight of 0.5);
- *e*: steepness of the growing curve of the logistic weighting function;
- *A*: minimum size of a two-dimensional spatial cluster to filter noise.

While the inclusion of a cell among the ones affected by drought is mostly dictated by the adopted drought indicator, the clustering algorithm divide/combine those cells into different events based on the adopted parameterization. In this context, the classical CDA approach is a special case of the adopted generalized approach, which can be obtained by searching for the closest contiguous cells (*L* = 3 and *p* = 0) and by adopting a simple Boolean approach to discriminate between drought and non-drought conditions (*k* = − 1 and *e* = 1000).

A range of different degrees of clustering can be obtained using different model settings^18^, hence the parameters of the clustering approach need to be tuned on target drought events. In this study, 38 documented past meteorological droughts are selected and characterized in terms of both temporal duration and spatial extent using an expert-based approach. The events are selected with the aim to cover different regions of the world, and to represent a large variety in terms of spatial extent and duration.

Starting from the same background information, and using only SPI-3 data as support, independent estimates by experts (from the team of the Copernicus European and Global Drought Observatories) are made for the main characteristics of each drought:

- Start date (year and month) marking the beginning of the event;
- End date (year and month) marking the conclusion of the event;
- Area interested by the drought (box extension encompassing the full area under drought between the start and the end dates).

These estimates are combined to obtain an ensemble of assessments, which is used to derive a reference dataset, based on the median of the ensemble (S3), as well as an estimate of the dispersion based on the 30-th and the 70-th percentiles (see S4,S5).

Overall, the expert assessments show large variability, with an average overlap between the 30-th and the 70-th percentile of about 50% and 60% in space and time, respectively, and a generally better agreement in the temporal domain compared to the spatial one (S6).

The exercise of building this reference dataset confirms the difficulty to assess univocally the full spatio-temporal evolution of a drought event, and it highlights how even experts can return rather different estimates starting from the same set of information. This uncertainty is accounted in the criteria adopted for the tuning of the algorithm, which is performed by using not only the ensemble median, but also the two percentiles.

As for the clustering algorithm, the outcomes for a given parameterisation can either overestimate the aggregation of drought cells – in space (larger clusters), or in time (longer events) – or underestimate the drought event (i.e. split an event into multiple sub-events). Given a reference condition (duration and spatial extent), the overestimation can be quantified by the ratio between the cells of the 3D cluster that fall inside the reference and the total size of the cluster (*f*_over_, equal to 1 when all the cells are inside the reference area). Analogously, the underestimation can be synthetically described by the fraction of cells under drought inside the reference that are not included in the given 3D cluster (*f*_under_, equal to 1 when all the drought cells in the reference are associated to the same event). More details on the performance of a parameterisation can be obtained by further disentangling these fractions, i.e. by discriminating spatial overestimates (cells in the right period but outside the expected extent) from temporal ones (cells in the right regions but before/after the expected period). However, these additional details, while useful to understand better the behaviour of one parameterization, do not play a major role in the overall assessment of the adherence of the model to the reference.

Both *f*_over_ and *f*_under_ can be computed with respect to the ensemble median, or against either the 30-th or the 70-th percentiles. In order to account for the uncertainty in the reference dataset, the two metrics are computed against all three percentiles, and the maximum value is selected for each event. Different sets of parameters are evaluated, and the optimal parameterization is chosen as the one that maximize the average of the two quantities (*f*_avg_), as a compromise between limiting the overestimation of the events (due to excessive linking among clusters) and reducing the over-fragmentation of the clusters.

## Supplementary Information

Supplementary Information.

## Data Availability

The SPI dataset used in this analysis can be retrieved from the GDO web portal (https://edo.jrc.ec.europa.eu/gdo/download). The codes used for the cluster analysis can be provided upon request through the GDO system.

## References

[1] United Nations Office for Disaster Risk Reduction (UNDRR). *GAR Special Report on Drought 2021*, (UNDRR, Geneva, 2021).

[2] Naumann G, Cammalleri C, Mentaschi L, Feyen L (2021). Increased economic drought impacts in Europe with anthropogenic warming. Nat. Clim. Change.

[3] Wood EF, Schubert SD, Wood AW, Peters-Lidard CD, Mo KC, Mariotti A, Pulwarty RS (2015). Prospects for advancing drought understanding, monitoring, and prediction. J. Hydrometeorol..

[4] Hao Z, Singh VP, Xia Y (2018). Seasonal drought prediction: Advances, challenges, and future prospects. Rev. Geophys..

[5] Lloyd-Hughes B (2012). A spatio-temporal structure-based approach to drought characterization. Int. J. Climatol..

[6] Herrera-Estrada JE, Satoh Y, Sheffield J (2017). Spatiotemporal dynamics of global drought. Geophys. Res. Lett..

[7] Zhou H, Liu Y, Liu Y (2019). An approach to tracking meteorological drought migration. Water Resour. Res..

[8] World Meteorological Organization (WMO) (2006). Drought Monitoring and Early Warning: Concepts, Progress, and Future Challenges.

[9] World Meteorological Organization (WMO) (2015). Seventeenth World Meteorological Congress: Abridged Final Report with Resolutions.

[10] Lackstrom, K., Brennan, A., Ferguson, D., Crimmins, M., Darby, L., Dow, K., Ingram, K., Meadow, A., Reges, H., Shafer, M. & Smith, K. The missing piece: Drought impacts monitoring. *Workshop report produced by the Carolinas Integrated Sciences & Assessments program and the Climate Assessment for the Southwest,* 5–6 March 2013, Tucson, AZ, 1-23, (2013).

[11] Stahl K, Kohn I, Blauhut V, Unquijo J, De Stefano L, Acácio V, Dias S, Stagge JH, Tallaksen LM, Kampragou E, Van Loon AF, Barker LJ, Melsen LA, Bifulco C, Musolino D, de Carli A, Massarutto A, Assimacopoulos D, Van Lanen HAJ (2016). Impacts of European drought events: Insights from an international database of text-based reports. Nat. Hazards Earth Syst. Sci..

[12] Mathbout S, Lopez-Bustins JA, Royé D, Martin-Vide J (2021). Mediterranean-scale drought: Regional datasets for exceptional meteorological drought events during 1975–2019. Atmosphere.

[13] González-Hidalgo JC, Vicente-Serrano SM, Peña-Angulo D, Salinas C, Tomas-Burguera M, Beguería S (2018). High-resolution spatio-temporal analyses of drought episodes in the western Mediterranean basin (Spanish mainland, Iberian Peninsula). Acta Geophys..

[14] Spinoni J, Naumman G, Vogt JV, Barbosa P (2015). The biggest drought events in Europe from 1950 to 2012. J. Hydrol. Reg. Stud..

[15] Spinoni J, Barbosa P, de Jager A, McCormick N, Naumann G, Vogt JV, Magni D, Masante D, Mazzaschi M (2019). A new global database of meteorological drought events from 1951 to 2016. J. Hydrol. Reg. Stud..

[16] Sheffield J, Andreadis KM, Wood EF, Lettenmaier DP (2009). Global and continental drought in the second half of the twentieth century: Severity-area-duration analysis and temporal variability of large scale events. J. Clim..

[17] Rakovec O, Samaniego L, Hari V, Markonis Y, Moravec V, Thober S, Hanel M, Kumar R (2022). The 2018–2020 multi-year drought sets a new benchmark in Europe. Earth Future.

[18] Cammalleri C, Toreti A (2023). A generalized density-based algorithm for the spatio-temporal tracking of drought events. J. Hydrometeorol..

[19] Rebetez M, Mayer H, Dupont O, Schindler D, Gartner K, Kropp JP, Menzel A (2003). Heat and drought 2003 in Europe: A climate synthesis. Ann. For. Sci..

[20] Ciais Ph, Reichstein M, Viovy N, Granier A, Ogée J, Allard V, Aubinet M, Buchmann N, Bernhofer Chr, Carrara A, Chevallier F, De Noblet N, Friend AD, Friedlingstein P, Grünwald T, Heinesch B, Keronen P, Knohl A, Krinner G, Loustau D, Manca G, Matteucci G, Miglietta F, Ourcival JM, Papale D, Pilegaard K, Rambal S, Seufert G, Soussana JF, Sanz MJ, Schulze ED, Vesala T, Valentini R (2005). Europe-wide reduction in primary productivity caused by the heat and drought in 2003. Nature.

[21] Toreti A, Belward A, Perez-Dominguez I, Naumann G, Luterbacher J, Cronie O, Seguini L, Manfron G, Lopez-Lozano R, Baruth B, van den Berg M, Dentener F, Ceglar A, Chatzopoulos T, Zampieri M (2019). The Exceptional 2018 European Water Seesaw calls for action on adaptation. Earth Future.

[22] Buras A, Rammig A, Zang CS (2020). Quantifying impacts of the drought 2018 on European ecosystems in comparison to 2003. Biogeosciences.

[23] Andreadis KM, Clark EA, Wood AW, Hamlet AF, Lettenmaier DP (2005). Twentieth-century drought in the conterminous United States. J. Hydrometeorol..

[24] Diaz V, Corzo Perez GA, Van Lanen HAJ, Solomatine D (2020). An approach to characterise spatio-temporal drought dynamics. Adv. Water Resour..

[25] Diaz V, Corzo Perez GA, Van Lanen HAJ, Solomatine D, Solomatine D, Corzo Perez GA (2022). Three-dimensional clustering in the characterization of spatiotemporal drought dynamics: Cluster size filter and drought indicator threshold optimization. Adv. Hydroinform: Artificial Intelligence and Optimization for Water Resources.

[26] Moravec V, Markonis Y, Rakovec O, Svoboda M, Trnka M, Kumar R, Hanel M (2021). Europe under multi-year droughts: How severe was the 2014–2018 drought period?. Environ. Res. Lett..

[27] Fleig AK, Tallaksen LM, Hisdal H, Hannah DM (2011). Regional hydrological drought in north-western Europe: Linking a new regional drought area index with weather types. Hydrol. Process..

[28] Stein U, Özerol G, Tröltzsch J, Landgrebe R, Szendrenyi A, Vidaurre R, Bressers H, Bressers N, Larrue C (2016). European drought and water scarcity policies. Governance for Drought Resilience.

[29] McKee, T.B., Doesken, N.J. & Kleist, J. The relationship of drought frequency and duration to time scales. In *8th Conference on Applied Climatology, Am. Meteorol. Soc.,* Boston, MC, 179–184 (1993).

[30] World Meteorological Organization (WMO) (2012). Standardized Precipitation index User Guide.

[31] Herbasch H (2020). The ERA5 global reanalysis. Q. J. R. Meteorol. Soc..

[32] Cammalleri C, Spinoni J, Barbosa P, Toreti A, Vogt JV (2021). The effects of non-stationarity on SPI for operational drought monitoring in Europe. Int. J. Climatol..

[33] World Meteorological Organization (WMO) (2017). WMO Guidelines on the Calculation of Climate Normals.

[34] Stasinopoulos DM, Rigby RA (2007). Generalized additive models for location scale and shape (GAMLSS). R. J. Stat. Softw..

[35] Ester, M., Kriegel, H.-P., Sander, J. & Xu, X. A density-based algorithm for discovering clusters in large spatial databases with noise. *KDD'96: Proc. of the 2nd International Conference on Knowledge Discovery and Data Mining* 226-231, 10.5555/3001460.3001507 (1996).

