# Supplementary material for: An event-oriented database of meteorological droughts in Europe based on spatio-temporal clustering
Source: Sci Rep. 2023 Feb 23;13:3145. doi: 10.1038/s41598-023-30153-6 (PMC9950368; doi:10.1038/s41598-023-30153-6)

Supplementary Information for: An event-oriented database of meteorological droughts in Europe based on spatio-temporal clustering

Carmelo Cammalleri, Juan Camilo Acosta Navarro, Davide Bavera, Vitali Diaz, Chiara Di Ciollo, Willem Maetens, Diego Magni, Dario Masante, Jonathan Spinoni & Andrea Toreti

S1. Detailed performance of the selected optimal algorithm (set = 2). The upper panel reports the complement to f_{over} splits between cells before the start of the event, after the end of the event and during the event but outside the domain. The lower panel reports the complement to f_{under}, representing the cells inside the area and during the period but that are considered part of other events.

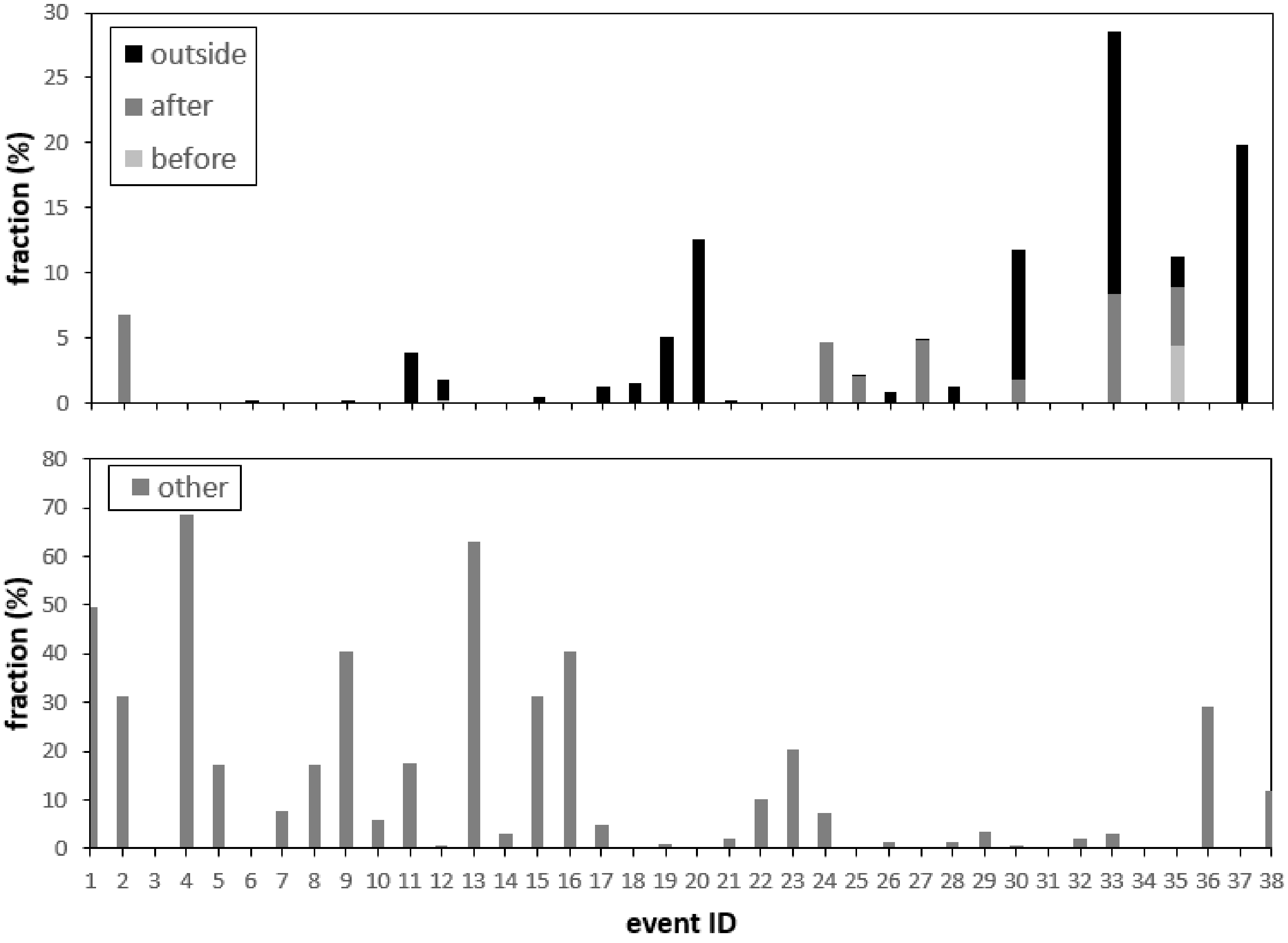

S2. Annual drought area according to the end-of-year SPI-12. Values are normalised as fraction of the total European domain. Drought categories are: moderate, SPI-12 < -1; severe, SPI-12 < -1.5; extreme, SPI-12 < -2.

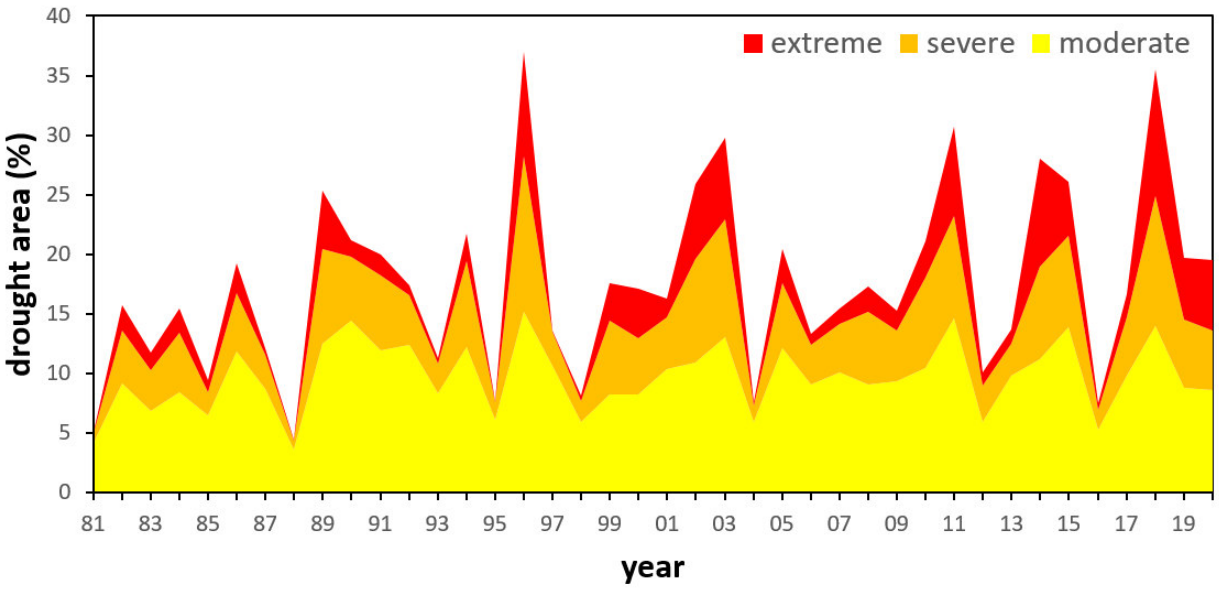

S3. Summary table on the characterization of the reference events according to the median of the ensemble. The fields 'description' and 'peak location' were provided to the experts to correctly identify the event. These data are just indicative of the location and the timing of the event. Upper-left (UL) corner and lower-right (LR) corner coordinates, as well as start and end dates, are derived from the ensemble as median.

ID	description	peak location		UL corner		LR corner		start		end	
		Y-M	lon, lat	lon	lat	lon	lat	Y	M	Y	M
1	Southern Asia 82/83	83-04	116, 1	92	22	132	-10	82	6	83	7
2	Sahel 83	83-07	-3, 7	-16	15	38	-4	83	1	83	12
3	NW USA 88	87-10	-121, 46	-127	59	-95	41	87	10	88	1
4	Argentina 89	89-01	-65, -28	-73	-22	-54	-51	88	10	89	7
5	Mediterranean 89/90	90-03	22, 41	4	48	38	34	89	12	90	5
6	Russia 91	91-05	67, 54	51	63	90	43	91	4	91	7
7	North Brazil 92	92-06	-58 ,1	-70	7	-38	-15	91	10	92	12
8	Southern Africa 91/92	92-02	32, -16	13	-7	41	-35	91	10	92	8
9	USA/Mexico 95/96	96-03	-100, 30	-124	42	-91	22	95	9	96	6
10	Southern Africa 94/95	95-01	20, -20	14	-2	37	-34	94	4	95	6
11	Iberian Peninsula 95	95-05	-6, 39	-10	44	4	33	94	12	95	6
12	Europe 95/96	96-01	9, 52	-7	70	44	47	95	12	96	7
13	Indonesia 97/98	97-10	111, -1	93	15	156	-10	97	1	98	6
14	USA/Mexico 98	98-06	-97, 31	-110	38	-79	12	98	1	98	8
15	Southwest Asia 2000	00-06	60, 36	35	43	74	20	00	1	00	8
16	Balkans 2000-2001	00-07	20, 46	14	50	35	35	00	4	01	2
17	Western USA 2002	02-06	-108, 39	-120	47	-95	30	02	1	02	9
18	India 2002	02-07	75, 29	65	33	85	7	02	7	02	11
19	Europe 2003	03-08	10, 50	-9	68	38	39	03	3	03	10
20	Eastern USA 2007	07-05	-88, 34	-93	40	-75	29	07	3	08	1
21	Eastern Australia 06/07	06-10	140, -33	127	-11	154	-43	06	6	07	2
22	Eastern Europe 06/07	07-01	22, 39	10	50	43	35	06	10	07	3
23	Horn of Africa 2008	08-04	39, 10	34	15	46	4.5	07	12	08	5
24	Argentina 2008	08-06	-59, -33	-67	-20	-53	-40	08	1	08	9
25	European Russia 2010	10-08	46, 54	33	63	83	44	10	4	10	10
26	Central USA 2012	12-07	-100, 38	-113	49	-82	32	12	1	12	12
27	Western Europe 2011	11-05	4, 51	-5	55	21	42	11	1	11	6
28	East China 2011	11-04	114, 28	97	39	122	21	10	12	11	11
29	Australia 2012	12-10	129, -29	113	-17	153	-39	12	4	13	2
30	Eastern Europe 2015	15-08	25, 51	2	58	43	43	15	6	15	11
31	Northeast Brazil 2016	16-04	-47, -11	-57	-3	-36	-23	16	4	16	7
32	South Africa 2016	15-12	25, -28	13	-10	37	-34	15	10	16	4
33	South India 2016	16-10	78, 12	73	18	83	6	16	8	17	1
34	Argentina 17/18	18-03	-63, -32	-70	-21	-55	-42	17	10	18	5
35	Mongolia 2019	19-02	110, 51	74	59	140	40	18	12	19	5
36	North Europe 2018	18-07	10, 54	-5	71	35	44	18	3	19	1
37	Southern Africa 18/19	19-01	24, -30	15	-16	35	-34	18	10	19	5
38	Australia 2019	19-12	150, -30	113	-12	154	-39	19	1	20	2

S4. Summary table on the characterization of the reference events according to the 30-th percentile of the ensemble. The fields ‘description’ and ‘peak location’ were provided to the experts to correctly identify the event. These data are just indicative of the location and the timing of the event. Upper-left (UL) corner and lower-right (LR) corner coordinates, as well as start and end dates, are derived from the ensemble as 30-th percentile.

ID	description	peak location		UL corner		LR corner		start		end	
		Y-M	lon, lat	lon	lat	lon	lat	Y	M	Y	M
1	Southern Asia 82/83	83-04	116, 1	91	24	163	-12	82	6	83	8
2	Sahel 83	83-07	-3, 7	-17	20	40	-5	83	1	83	12
3	NW USA 88	87-10	-121, 46	-128	60	-89	40	87	10	88	2
4	Argentina 89	89-01	-65, -28	-76	-20	-50	-53	88	6	89	7
5	Mediterranean 89/90	90-03	22, 41	-10	51	44	32	89	10	90	9
6	Russia 91	91-05	67, 54	48	64	97	42	91	3	91	7
7	North Brazil 92	92-06	-58 ,1	-77	8	-35	-21	91	10	93	6
8	Southern Africa 91/92	92-02	32, -16	12	-6	46	-35	91	6	92	8
9	USA/Mexico 95/96	96-03	-100, 30	-125	45	-90	20	95	6	96	7
10	Southern Africa 94/95	95-01	20, -20	12	-2	41	-35	93	9	95	7
11	Iberian Peninsula 95	95-05	-6, 39	-11	45	4.5	29	94	12	95	7
12	Europe 95/96	96-01	9, 52	-11	70	102	40	95	12	96	8
13	Indonesia 97/98	97-10	111, -1	92	20	163	-14	97	1	98	9
14	USA/Mexico 98	98-06	-97, 31	-110	40	-74	10	98	1	98	9
15	Southwest Asia 2000	00-06	60, 36	34	47	79	16	99	12	00	9
16	Balkans 2000-2001	00-07	20, 46	13	51	47	35	00	4	01	2
17	Western USA 2002	02-06	-108, 39	-127	50	-95	28	02	1	02	12
18	India 2002	02-07	75, 29	65	33	88	6	02	6	02	12
19	Europe 2003	03-08	10, 50	-11	70	50	37	03	1	03	11
20	Eastern USA 2007	07-05	-88, 34	-95	44	-75	26	07	1	08	2
21	Eastern Australia 06/07	06-10	140, -33	127	-10	154	-43	06	1	07	2
22	Eastern Europe 06/07	07-01	22, 39	9	50	45	34	06	10	07	8
23	Horn of Africa 2008	08-04	39, 10	33	16	48	4	07	12	08	5
24	Argentina 2008	08-06	-59, -33	-70	-19	-51	-50	07	12	08	9
25	European Russia 2010	10-08	46, 54	30	65	93	40	10	4	10	10
26	Central USA 2012	12-07	-100, 38	-126	53	-77	20	11	9	12	12
27	Western Europe 2011	11-05	4, 51	-6	57	22	41	11	1	11	6
28	East China 2011	11-04	114, 28	96	40	124	20	10	12	11	12
29	Australia 2012	12-10	129, -29	113	-16	154	-39	12	4	13	4
30	Eastern Europe 2015	15-08	25, 51	1	60	45	41	15	4	15	11
31	Northeast Brazil 2016	16-04	-47, -11	-70	0	-35	-23	15	9	16	8
32	South Africa 2016	15-12	25, -28	13	-9	45	-35	15	10	16	4
33	South India 2016	16-10	78, 12	72	18	83	6	16	7	17	1
34	Argentina 17/18	18-03	-63, -32	-72	-20	-52	-55	17	10	18	5
35	Mongolia 2019	19-02	110, 51	72	60	140	37	18	12	19	5
36	North Europe 2018	18-07	10, 54	-11	72	35	43	18	1	19	2
37	Southern Africa 18/19	19-01	24, -30	13	-15	36	-35	18	10	20	1
38	Australia 2019	19-12	150, -30	113	-11	154	-41	18	12	20	2

S5. Summary table on the characterization of the reference events according to the 70-th percentile of the ensemble. The fields 'description' and 'peak location' were provided to the experts to correctly identify the event. These data are just indicative of the location and the timing of the event. Upper-left (UL) corner and lower-right (LR) corner coordinates, as well as start and end dates, are derived from the ensemble as 70-th percentile.

ID	description	peak location		UL corner		LR corner		start		end	
		Y-M	lon, lat	lon	lat	lon	lat	Y	M	Y	M
1	Southern Asia 82/83	83-04	116, 1	98	13	128	-6	82	10	83	5
2	Sahel 83	83-07	-3, 7	-14	13	35	0	83	1	83	6
3	NW USA 88	87-10	-121, 46	-127	53	-100	43	87	10	88	1
4	Argentina 89	89-01	-65, -28	-69	-23	-61	-43	88	10	89	4
5	Mediterranean 89/90	90-03	22, 41	12	44	36	37	90	1	90	5
6	Russia 91	91-05	67, 54	54	60	89	44	91	4	91	7
7	North Brazil 92	92-06	-58 ,1	-69	5	-38	-12	92	4	92	9
8	Southern Africa 91/92	92-02	32, -16	18	-9	38	-32	91	10	92	7
9	USA/Mexico 95/96	96-03	-100, 30	-122	42	-95	23	95	10	96	5
10	Southern Africa 94/95	95-01	20, -20	15	-5	34	-32	94	9	95	6
11	Iberian Peninsula 95	95-05	-6, 39	-9	43	2	35	94	12	95	6
12	Europe 95/96	96-01	9, 52	-3	68	41	47	95	12	96	7
13	Indonesia 97/98	97-10	111, -1	95.5	8	148	-10	97	1	98	5
14	USA/Mexico 98	98-06	-97, 31	-108	36	-88	17	98	4	98	8
15	Southwest Asia 2000	00-06	60, 36	40	42	68	25	00	1	00	7
16	Balkans 2000-2001	00-07	20, 46	15	49	28	37	00	4	01	1
17	Western USA 2002	02-06	-108, 39	-119	44	-95	32	02	1	02	9
18	India 2002	02-07	75, 29	65	32	80	22	02	7	02	10
19	Europe 2003	03-08	10, 50	0	54	24	43	03	3	03	10
20	Eastern USA 2007	07-05	-88, 34	-92	38	-78	29	07	3	07	9
21	Eastern Australia 06/07	06-10	140, -33	129	-14	152	-42	06	7	07	1
22	Eastern Europe 06/07	07-01	22, 39	13	46	41	37	06	10	07	2
23	Horn of Africa 2008	08-04	39, 10	36	13	44	5	08	1	08	5
24	Argentina 2008	08-06	-59, -33	-66	-22	-54	-38	08	4	08	8
25	European Russia 2010	10-08	46, 54	34	59	65	46	10	6	10	10
26	Central USA 2012	12-07	-100, 38	-110	45	-83	32	12	3	12	11
27	Western Europe 2011	11-05	4, 51	-5	55	19	43	11	3	11	6
28	East China 2011	11-04	114, 28	99	35	121	21	10	12	11	10
29	Australia 2012	12-10	129, -29	114	-20	150	-36	12	6	13	2
30	Eastern Europe 2015	15-08	25, 51	12	58	40	45	15	6	15	10
31	Northeast Brazil 2016	16-04	-47, -11	-57	-4	-38	-21	16	4	16	7
32	South Africa 2016	15-12	25, -28	14	-12	33	-33	15	10	16	2
33	South India 2016	16-10	78, 12	74	17	82	6	16	9	17	1
34	Argentina 17/18	18-03	-63, -32	-67	-25	-56	-39	17	10	18	4
35	Mongolia 2019	19-02	110, 51	84	57	131	42	18	12	19	4
36	North Europe 2018	18-07	10, 54	-2	68	30	47	18	3	19	1
37	Southern Africa 18/19	19-01	24, -30	15	-17	32	-34	18	10	19	3
38	Australia 2019	19-12	150, -30	114	-13	154	-38	19	6	20	1

S6. Overlap between the 30-th and 70-th percentile reference datasets for area (left panel) and duration (right panel).

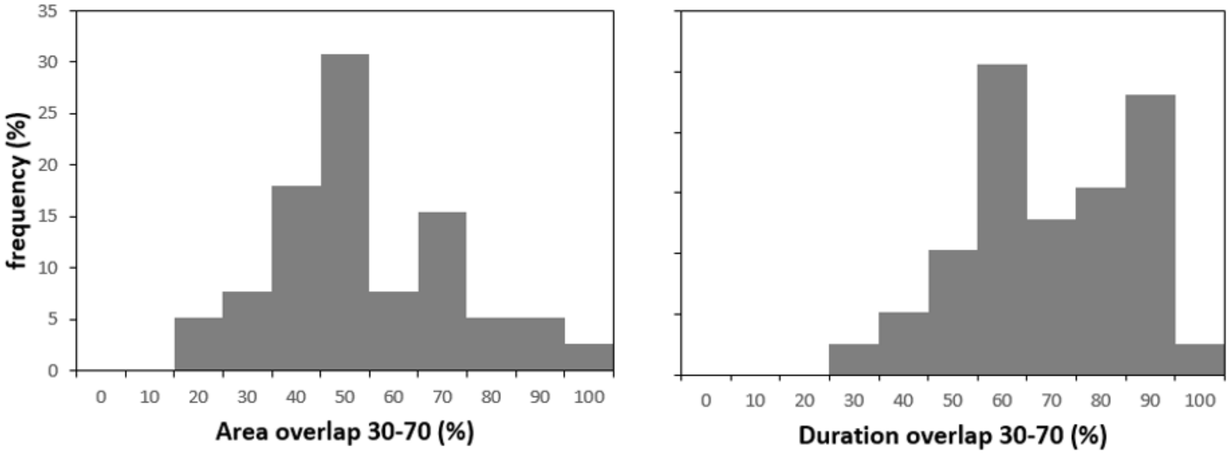

Supplement: Supplementary file 1 — Supplementary Information. [file 41598_2023_30153_MOESM1_ESM.pdf]
